# Semaphorin 7a aggravates TGF-β1-induced airway EMT through the FAK/ERK1/2 signaling pathway in asthma

**DOI:** 10.3389/fimmu.2023.1167605

**Published:** 2023-11-01

**Authors:** Haiying Peng, Fei Sun, Yunxiu Jiang, Zihan Guo, Xinyi Liu, Anli Zuo, Degan Lu

**Affiliations:** Department of Respiratory, The First Affiliated Hospital of Shandong First Medical University & Shandong Provincial Qianfoshan Hospital, Shandong Institute of Respiratory Diseases, Shandong Institute of Anesthesia and Respiratory Critical Medicine, Jinan, China

**Keywords:** asthma, semaphorin 7A, airway remodeling, epithelial-mesenchymal transition, TGF-β1

## Abstract

**Background:**

TGF-β1 can induce epithelial-mesenchymal transition (EMT) in primary airway epithelial cells (AECs). Semaphorin7A (Sema7a) plays a crucial role in regulating immune responses and initiating and maintaining transforming growth factor β1 TGF-β1-induced fibrosis.

**Objective:**

To determine the expression of Sema7a, in serum isolated from asthmatics and non-asthmatics, the role of Sema7a in TGF-β1 induced proliferation, migration and airway EMT in human bronchial epithelial cells (HBECs) *in vitro*.

**Methods:**

The concentrations of Sema7a in serum of asthmatic patients was detected by enzyme-linked immunosorbent assay (ELISA). The expressions of Sema7a and integrin-β1 were examined using conventional western blotting and real-time quantitative PCR (RT-PCR). Interaction between the Sema7a and Integrin-β1 was detected using the Integrin-β1 blocking antibody (GLPG0187). The changes in EMT indicators were performed by western blotting and immunofluorescence, as well as the expression levels of phosphorylated Focal-adhesion kinase (FAK) and Extracellular-signal-regulated kinase1/2 (ERK1/2) were analyzed by western blot and their mRNA expression was determined by RT-PCR.

**Results:**

We described the first differentially expressed protein of sema7a, in patients with diagnosed bronchial asthma were significantly higher than those of healthy persons (P<0.05). Western blotting and RT-PCR showed that Sema7a and Integrin-β1 expression were significantly increased in lung tissue from the ovalbumin (OVA)-induced asthma model. GLPG0187 inhibited TGF-β1-mediated HBECs EMT, proliferation and migration, which was associated with Focal-adhesion kinase (FAK) and Extracellular-signal-regulated kinase1/2 (ERK1/2) phosphorylation.

**Conclusion:**

Sema7a may play an important role in asthma airway remodeling by inducing EMT. Therefore, new therapeutic approaches for the treatment of chronic asthma, could be aided by the development of agents that target the Sema7a.

## 1 Introduction

Asthma, characterized by airway inflammation, airway hyperresponsiveness (AHR), and airway remodeling (AR), is a chronic and heterogenic disease of the respiratory system mainly due to occupational or environmental exposure to industrial products, microorganisms, and other allergens (1, 2). The prevalence of asthma is still on the rise, with an estimated 358 million people worldwide affected by asthma, according to a global burden of disease study in 2015 (3). Most asthmatic patients can be controlled with bronchodilators and inhaled corticosteroids (4). However, an estimated 5-10% of patients are refractory to the treatment and thus require further therapy and even hospitalization, which results in impaired quality of life and a disproportionate cost to healthcare systems (5). ​AR may play an important role in the clinical severity of refractory asthma (6, 7). Therefore, it is necessary to further understand the factors that regulate the pathological features of asthma, including chronic inflammation and AR.

The chronic inflammatory response in the airways of asthmatic patients may result in alterations in the composition and distribution of cellular constituents of the airway wall, which is termed AR (8, 9). As a key feature of asthma, AR leads to irreversible airflow obstruction and persistent airway hyperresponsiveness and contributes to the symptomatology of the disease as well as irreversible loss of lung function (10, 11). Currently, inhaled corticosteroids and long-acting β2 agonists remain the mainstay for guideline-based control and management for asthma (12). However, such therapeutics are neither proven to prevent nor reverse AR although they can ameliorate inflammation (13). Moreover, AR may occur early in childhood to an equivalent degree in the airways, not necessarily subsequent to inflammation (14). Therefore, it is necessary to further explore the pathogenesis of AR in allergic asthma.

The semaphorin (Sema) protein family consists of more than 20 members and are extracellular signaling proteins that are instrumental in the development and maintenance of several organs and tissues (15). As a member of the Sema family, Sema7a (also known as CD108) is a glycosylphosphatidylinositol-linked membrane protein and is expressed constitutively and broadly in a variety of lymphoid, bone, endothelial, and nerve cells (16, 17). By binding to its receptor, integrin-β1 or plexin C1, with high affinity, Sema7a stimulates cytokine production in some inflammatory cells and is essential to the effector phase of the inflammatory immune response (18–20). The interaction of Sema7a with its receptor activates multiple signaling pathways, including phosphorylated focal-adhesion kinase phosphorylated (p-FAK), phosphorylated extracellular-signal-regulated kinase 1/2 (p-ERK1/2), nuclear factor kappa B (NF-κB), transforming growth factor β2 (TGF-β2)/Smad, and others (21, 22). A battery of studies has found that the Sema7a-integrin-β1 axis is a pivotal pathway in cell migration, angiogenesis and endothelial damage and is implicated in some disorders (22–24). Nevertheless, whether Sema7a has a role in the development of allergic asthma remains obscure.

Epithelial-mesenchymal transformation (EMT) is characterized by epithelial cell damage–repair-redamage-repair and is considered to be an initiating factor in AR in asthma (25, 26). As the first barrier to contact allergens, the bronchial epithelium plays an important role in airway EMT by secreting a variety of proinflammatory factors (27). Many signaling pathways, including transforming growth factor (TGF-β), epidermal growth factor (EGF), and tumor necrosis factor (TNF-α), are involved in the EMT process (28). Among them, TGF-β1 is a major inducer of EMT (29, 30). When expressed in the pulmonary epithelium, TGF-β1 can lead to the development of several AR features, including subepithelial fibrosis, epithelial shedding, and extracellular matrix deposition in the subepithelial layer by inducing EMT in bronchial epithelial cells (29, 31). In primary airway epithelial cells (AECs) obtained from asthmatic patients, TGF-β1 can induce EMT in a Smad3-dependent manner, suggesting dysregulated epithelial repair in asthmatic airways (32). Although some studies have documented that Sema7a contributes to atherosclerosis by mediating endothelial dysfunction and promotes the growth and migration of oral tongue squamous carcinoma cells by regulating the course of EMT (21, 33), little is known about whether Sema7a is involved in AR in allergic asthma by promoting the process of EMT.

To sum up, the expressions of Sema7a in serum of asthmatic patients and in lung tissue of ovalbumin (OVA) -induced mice were first examined. Then, whether the expression of Sema7a was associated with pathological features of allergic asthma was evaluated. Next, the roles of Sema7a in TGF-β1-induced EMT in human bronchial epithelial cells (HBECs) were investigate. Finally, the main signaling pathways involved in the effect of Sema7a were further probed. Our findings suggest that Sema7a may play a fundamental role in AR by inducing EMT through the FAK and ERK1/2 signaling pathway in HBECs and mice model of chronic asthma.

## 2 Methods

### 2.1 Humans

​ Ten patients with asthma were consecutively recruited from the Department of Respiratory Medicine and Intensive Care Medicine at the First Affiliated Hospital of Shandong First Medical University between October 1, 2021, and April 30, 2022. All of them met the criteria for asthma described in the report of the Global Initiative for Asthma (34). The disease activity of asthma was assessed according to the Asthma Control Test (ACT). Serum samples were collected, and those whose percentage of eosinophils in peripheral blood exceeded 3% were enrolled. A total of 20 healthy individuals who underwent health check-up in our hospital and whose age and sex were matched with those of asthmatic patients were selected as controls. None of them have chronic airway diseases/cancer or autoimmune diseases. This study was approved by the Medical Ethics Committee of The First Affiliated Hospital of Shandong First Medical University. Informed consent was obtained from all subjects who were enrolled in the present study.

### 2.2 Mice

A total of 18 female BALB/c mice aged 6-8 weeks were purchased from Pengyue Experimental Animal Breeding Co. Ltd. (Jinan, China) and housed in the Laboratory Animal Center of our hospital under SPF conditions. The feeding environment of the mice included a temperature−controlled house with 12 h light−dark cycles and free access to a standard laboratory diet and water (35). The mice were randomly divided into 3 groups: the control group (control, n=6), asthma group (asthma, n=6) and anti-Sema7a group (anti-Sema7a, n=6). All animal procedures were performed according to the National Institutes of Health (NIH) Guide for the Care and Use of Laboratory Animals (36). Moreover, all protocols were approved by the Ethics Committee for Laboratory Animals Care and Use in First Affiliated Hospital of Shandong First Medical University, Shandong, China.

### 2.3 Experimental model of chronic asthma

**Figure 1:**
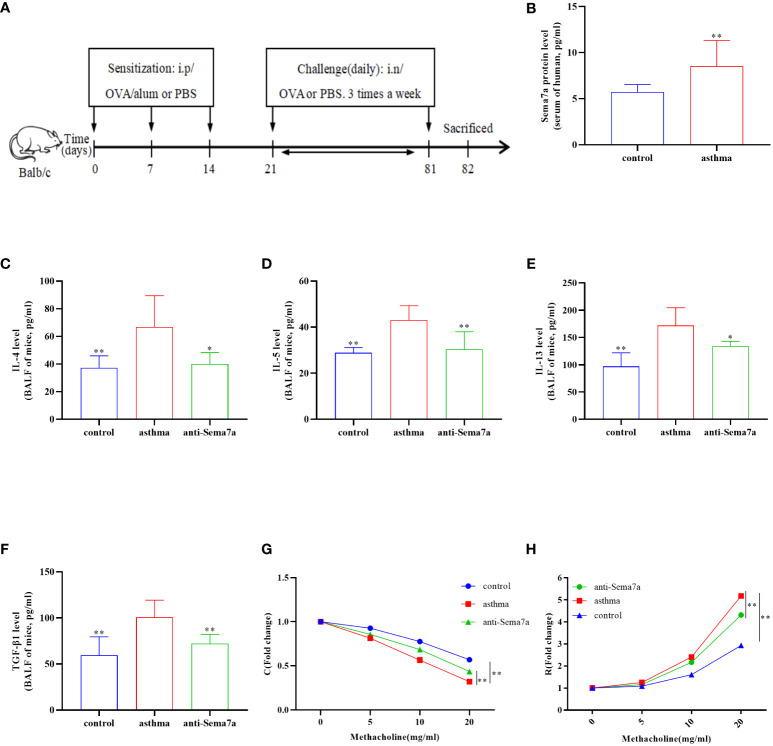
Airway inflammation and AHR in an OVA-challenged mouse model. **(A)** The establishment of chronic asthma mouse model; **(B)** The level of Sema7a in serum of patients with asthma was higher than that in serum of healthy subjects. **(C)** The asthma group had higher IL-4 levels in the BALF than the control group and the anti-Sema7a group had lower IL-4 levels than the asthma group. **(D)** The asthma group had higher IL-5 levels in the BALF than the control group and the anti-Sema7a group had lower IL-4 levels than the asthma group. **(E)** The asthma group had higher IL-13 levels in the BALF than the control group and the anti-Sema7a group had lower IL-4 levels than the asthma group. **(F)** The asthma group had higher TGF-B 1 levels in the BALF than the control group and the anti-Sema7a group had lower IL-4 levels than the asthma group. **(G)** It does not reveal significant differences among the three groups of baseline airway responsiveness (at 0 mg/ml methacholine); Stimulated by acetylcholine, the C was significantly descended in asthma group compared with control group, while enhanced in anti-Sema7a group compared with asthma group. **(H)** Stimulated by acetylcholine, the R was significantly enhanced in asthma group compared with control group, while descended in anti-Sema7a group compared with asthma group. Data represent means ± SD. ^*^P < 0.05, ^**^P < 0.01.

As described previously (37), the asthma group was sensitized with 0.2 ml sensitinogen (20 µg OVA+2 mg aluminum hydroxide) (Sigma−Aldrich, Beijing, China) on days 1, 7 and 14. From day 21 on, the OVA-sensitized mice were exposed to aerosolized 3% OVA (Sigma−Aldrich, Beijing, China) after anesthesia with 1% sodium pentobarbital (38) in a 30 cm×24 cm×50 cm chamber for 8 consecutive weeks: three times a week, 30 min each time. Anti-Sema7a antibody (6 µg/mouse) (AF1835, goat IgG; R&D Systems, Minneapolis, MN, USA) was administered intratracheally 30 min before each of the antigen challenges. In addition, mice in the control group were sensitized and challenged with phosphate-buffered saline (PBS) alone (**Figure 1A**).

### 2.4 Measurement of allergen-induced AHR

Airway responsiveness (R) and dynamic compliance (C) to methacholine challenge were evaluated 24 h following the last OVA challenge as previously reported (39). Briefly, mice were anesthetized with 0.12-0.15 ml 1% pentobarbital sodium intraperitoneally (22, 40). After the mice were fully anesthetized, a cannula was subsequently inserted into the trachea, and the mice were connected to the flexiVent system (Scireq). Subsequently, the mice were mechanically ventilated at a rate of 150 breaths/min, a tidal volume of 5 mL/kg, and a positive-end expiratory pressure of 3 cm H_2_O (35). They were initially challenged with saline followed by challenge with increasing concentrations of methacholine (0, 5, 10, and 20 mg/ml; Sigma−Aldrich; Merck KGaA) for 10 sec at each dose. R and C were calculated as percentage increases over baseline (saline challenge).

### 2.5 Enzyme-linked immunosorbent assay

Mice were euthanized by cervical vertebrae dislocation immediately after lung function measurement (41). Right-lung lavage was performed with 2 ml PBS, and bronchoalveolar lavage fluid (BALF) was collected and centrifuged at 4°C and 80xg for 10 min. The concentrations of IL-4 (SEA077Mu), IL-5 (SEA078Mu), IL-13 (SEA060Mu) and TGF-β1 (SEA124Mu) in the BALF were determined using ELISA kits (Cloud-Clone, Wuhan, China) according to the manufacturer’s protocol.

### 2.6 Histological analysis

The left lungs of mice were fixed in 4% paraformaldehyde at room temperature for 24 h (42). Then, the lung tissues were embedded in paraffin and cut into 5-μm-thick sections. Inflammatory infiltration and airway wall thickness were evaluated by hematoxylin and eosin (H&E) staining. Collagen deposition within the mouse airway wall was assessed by Masson’s trichrome staining (Masson). To assess goblet cell proliferation in the airway wall, the sections were stained with periodic acid-Schiff stain (PAS) (35).

Before staining, serial 5-μm-thick sections were dewaxed in xylene and rehydrated through a series of decreasing concentrations of ethanol (43). Sections were placed in sodium citrate (pH 6.0, G1201-1 L, Servicebio, Wuhan, China), boiled for 10 minutes, and cooled to room temperature for antigen retrieval. The appropriate amount of endogenous peroxidase blocker was added to the sample, which was incubated at room temperature in the dark for 10 min and then rinsed with PBS. The sections were treated with goat serum (SP-9001, Zhongshan, Beijing, China) at room temperature for 15 min to block nonspecific binding and incubated overnight at 4°C with rabbit polyclonal antibody against Sema7a (1:400, bs-2702R, Bioss, Beijing, China) diluted in PBS. After washing in PBS three times (5 min each time), the sections were incubated with anti-rabbit IgG antibody conjugated to horseradish peroxidase (HRP) (SP-9001, Zhongshan, Beijing, China). Next, DAB chromogen solution was added to the slide for 5 min at room temperature, washed extensively with PBS, and counterstained with hematoxylin for 20 s. Finally, slices were dehydrated with a gradient alcohol series, cleared in xylene and sealed with neutral gum. All sections were observed using a BX51 microscopic imaging system (Olympus Corporation, Japan).

### 2.7 Cell culture

The human bronchial epithelial cell line (16HBECs) was obtained from Fuheng Biology (Shanghai, China) and cultured in keratinocyte culture medium (KM) and penicillin−streptomycin. 16HBECs were treated with different concentrations of TGF-β1 (2.5, 5 and 10 ng/ml) for 12 h, 24 h or 48 h with or without pretreatment with integrin-β1 blockers (GLPG0187) (2 µm, 5 μm, 10 µm) for 1 h. The cells were harvested for the following experiments.

### 2.8 Cell proliferation assay

Cells were detached from the 25 cm3 cell-culture flask with 0.25% trypsin-EDTA (1×) (25200-056, Gibco, Thermo Scientific), and they were counted and suspended at a density of 5×10^5^/ml in KM culture medium. Then, cells were seeded onto 96-well plates at a density of 2000 cells/well for 24 h and subsequently incubated with KM culture medium containing various dilutions of TGF-β1 and integrin-β1 (0 ng/ml TGF-β1 + 0 µm GLPG0187, 5 ng/ml TGF-β1 + 0 µm GLPG0187, 5 ng/ml TGF-β1 + 2 µm GLPG0187, 5 ng/ml TGF-β1 + 5 µm GLPG0187, 5 ng/ml TGF-β1 + 10 µm GLPG0187) at 37°C in a 5% CO_2_ humidified atmosphere for 12, 24, 48 and 72 h. Following incubation for the indicated times, 10 µl Cell Counting Kit-8 (CCK-8) solution was added to each well and incubated for 2 h at 37°C with 5% CO_2_ to examine the effect of integrin-β1 on 16HBECs proliferation. Cell proliferation was determined by measuring the absorbance at 450 nm using a microplate spectrophotometer.

### 2.9 Reverse transcription−quantitative PCR

Total RNA in mouse lung tissues was extracted using an RNA Isolation Kit (RC101, Vazyme, Nanjing, China) according to the manufacturer’s instructions. First-strand cDNA was generated from 1 µg of total RNA using an RT SuperMix RT kit (R323, Vazyme, Nanjing, China) to prime the reverse transcription reaction according to the manufacturer’s protocol. Gene expression was determined by SYBR Real-Time PCR Master Mix (Q711, Vazyme, Biotechnology). The primer pairs were synthesized by Takara (**Table 1**). Target mRNA levels were determined using the quantification cycle (Cq) values normalized against the expression of GAPDH. Gene expression was calculated using the 2^-ΔΔcq^ method (44).

**Table 1:** Primer sequences for the target genes.

Target gene	Primer sequence (5’→3’)
Mice Sema7a forward	TACCAGGGTCTATGGCGTTTTC
Mice Sema7a reverse	GCCCATGTGGTAGCCTTTGA
Mice Integrin-β1 forward	GGGTATTT GTGAATGTGGTGCTT
Mice Integrin-β1 reverse	TTTGGTGAGATTGAAGTGGGAGC
Mice GAPDH forward	AGAAACCTGCCAAGTATGATGACA
Mice GAPDH reverse	GGAAGAGTGGGAGTTGCTGTTG

### 2.10 Western blot analysis

The tissue and cell proteins were extracted by using RIPA buffer, phenylmethanesulfonyl fluoride (PMSF), and phosphatase inhibitors. Protein concentration was determined with a protein assay kit (P0012; Beyotime Biotechnology, Shanghai, China) according to the protocol described previously (43). The samples were separated by 7.5-10% sodium dodecyl sulfate−polyacrylamide gel electrophoresis (SDS−PAGE), and the bands were electrotransferred to a 0.45 mm polyvinylidene fluoride (PVDF) membrane. After blocking with 5% skim milk, the membrane was incubated at 4°C overnight with rat monoclonal antibody for Sema7a (1:500, MAB2068, R&D Systems), rabbit monoclonal antibody for integrin-β1 (1:2,000, ab179471, Abcam), mouse monoclonal antibody for E-cadherin (1:1,000, ab231303, Abcam), rabbit monoclonal antibody for fibronectin (1:1,000, ab268020, Abcam), α-SMA (1:10,000, ab124964, Abcam), focal-adhesion kinase (FAK) (1:500, SAB4502495, Sigma), extracellular-signal-regulated kinase1/2 (ERK1/2) (1:20,000, M5670, Sigma), p-FAK (1:1000, ab81298, Abcam), and p-ERK1/2 (1:1000, SAB4301578, Sigma). The secondary antibody was incubated at room temperature for 1 h and washed with PBS three times. An enhanced chemiluminescence detection system was used to detect target proteins, with β-tubulin as the loading control. All data were analyzed by ImageJ Software (version 10.4, Tree Star Inc.).

### 2.11 Statistical analysis

All data are expressed as the means ± standard deviations (SD). The results were analyzed using GraphPad Prism 8 software. Pairwise comparisons were performed using Student’s t test, and comparisons among multiple groups were conducted using one-way analysis of variance. Values of P < 0.05 indicated statistical significance.

## 3 Results

### 3.1 Expression of Sema7a in serum was enhanced significantly in asthmatic patients

Previous studies have found that Sema7a is expressed on airway eosinophils and plays an important role in immunoglobulin-E (Ig-E)-mediated airway inflammation (45, 46). Therefore, we examined whether Sema7a expression in serum differs between allergic asthmatic individuals and healthy individuals. As shown in **Figure 1B**, the level of Sema7a in patients with asthma was significantly higher than that in healthy subjects (**Figure 1B**; *P* < 0.01). These findings suggest that Sema7a is likely implicated in the development of allergic asthma.

### 3.2 Expression of Sema7a and integrin-β1 in lung tissue was obviously increased in OVA-treated mice

**Figure 2:**
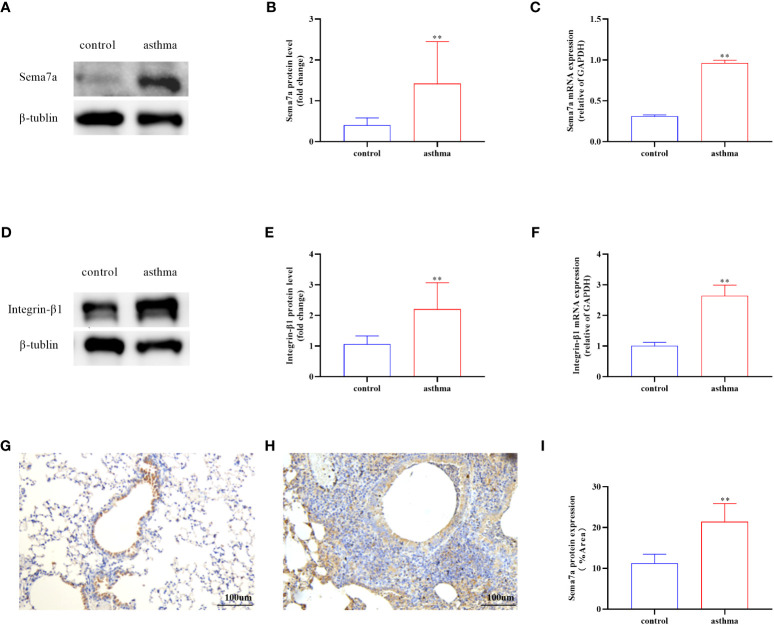
Protein and mRNA expression in lung tissues of Sema7a and Integrin-β1 in each group. **(A, B)** The level of Sema7a protein in lung tissue of asthma group was higher than control group. **(C)** The level of Sema7a mRNA in lung tissue of asthma group was higher than control group. **(D, E)** The level of Integrin-β1 protein in lung tissue of asthma group was higher than control group. **(F)** The level of Integrin-β1 mRNA in lung tissue of asthma group was higher than control group. **(G–I)** Immunohistochemical analyses revealed that the expression of Sema7a in asthmatic epithelial cells was significantly higher than that in the control group. Data represent means ± SD.^**^P < 0.01.

Sema7a is preferentially expressed on activated T cells, eosinophils, and thymocytes (47–49). The Sema7a gene is moderately expressed in the heart, lung, and pancreas in mice. Sema7a can mediate macrophage and dendritic cell migration and tumor lymphatic metastasis through integrin-β1 receptors (50). We asked whether the expression status of Sema7a and integrin-β1 protein is different between PBS-treated mice and OVA-treated mice. To this aim, we established a chronic asthmatic mouse model. As shown in **Figures 2A, D**, Sema7a and integrin-β1 were both expressed in lung tissue in mice. Sema7a and integrin-β1 expression were significantly higher than those of the control group (**Figures 2B, C, E, F**; *P* < 0.01). Although Sema7a expression was observed in bronchial epithelial cells of both asthmatic and control mice, immunohistochemical analyses revealed that the expression of Sema7a in asthmatic epithelial cells was significantly higher than that in the control group (**Figures 2G–I**; *P* < 0.01). These findings indicate that Sema7a is likely involved in the pathogenesis of allergic asthma.

### 3.3 AHR and AR were alleviated by Sema7a blockade in a chronic asthmatic model

**Figure 3:**
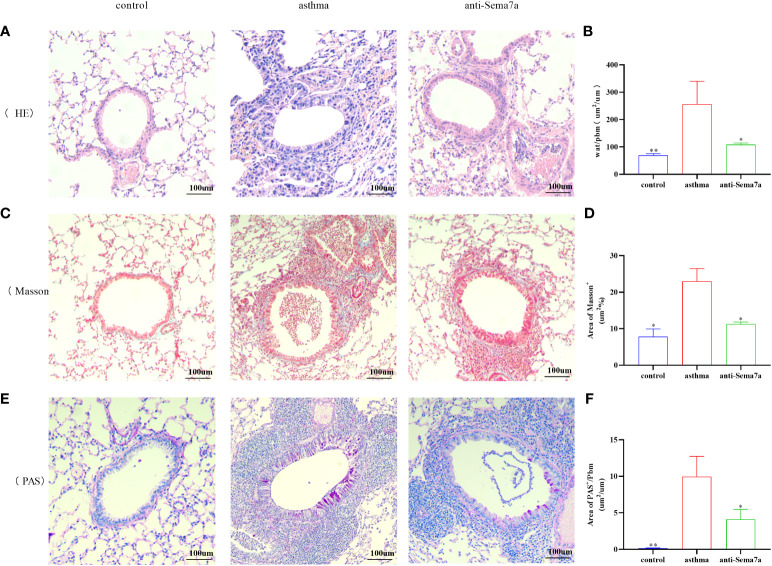
Histological examination of lung tissue was performed 24 h after the final OVA challenge. Lung tissues were fixed, sectioned at 5 um thickness, and stained with hematoxylin-eosin (HE), periodic acid-Schiff (PAS), and Masson stain. **(A, B)** Quantified results of airway wall thickness (Wat) analyzed by Image-Pro® Plus 6.0 software. **(C, D)** Quantified results of airway wall thickness (Wat) analyzed by Image-Pro® Plus 6.0 software. **(E, F)** Quantitative analysis of collagen deposition by Image-Pro® Plus 6.0 software. Data represent means ± SD. ^*^P < 0.05, ^**^P < 0.01.

AR is one of the key features of asthma and has an essential role in disease progression (51), which often correlates with the severity of clinical disease (52, 53). AR is regarded as an important factor contributing to AHR and irreversible airflow limitation (54–56). In the present study, the association between Sema7a and AHR and AR was subsequently probed. As illustrated in **Figures 1G, H**, there were no significant differences among the three groups of baseline airway responsiveness (at 0 mg/ml methacholine) (**Figures 1G, H**; *P* > 0.05). Stimulated by acetylcholine, R was significantly enhanced and C was decreased in the asthma group compared with the control group (**Figures 1G, H**; *P* < 0.01), while R was significantly decreased and C was enhanced in the anti-Sema7a group mice compared with the asthma group mice (**Figures 1G, H**; *P* < 0.01). In addition, the ELISA results showed that the levels of these inflammatory factor cytokines in serum were significantly decreased in anti-Sema7a group mice compared with the OVA-challenged group (**Figures 1C–F**; *P* < 0.05). At 200× objectives with H&E staining, the accumulation of airway inflammatory factors, turbulence of bronchial epithelial cells, stenosis of the bronchial cavity, and thickening of the airway wall were observed in OVA-challenged mice. All of the abovementioned pathological changes were alleviated by preadministration of anti-Sema7a, as shown in **Figure 3**. These findings clearly suggest that Sema7a and integrin-β1 play a role in the process of AR in a mouse model of allergic asthma.

### 3.4 Sema7a and integrin-β1 were induced by TGF-β1 in 16HBECs

**Figure 4:**
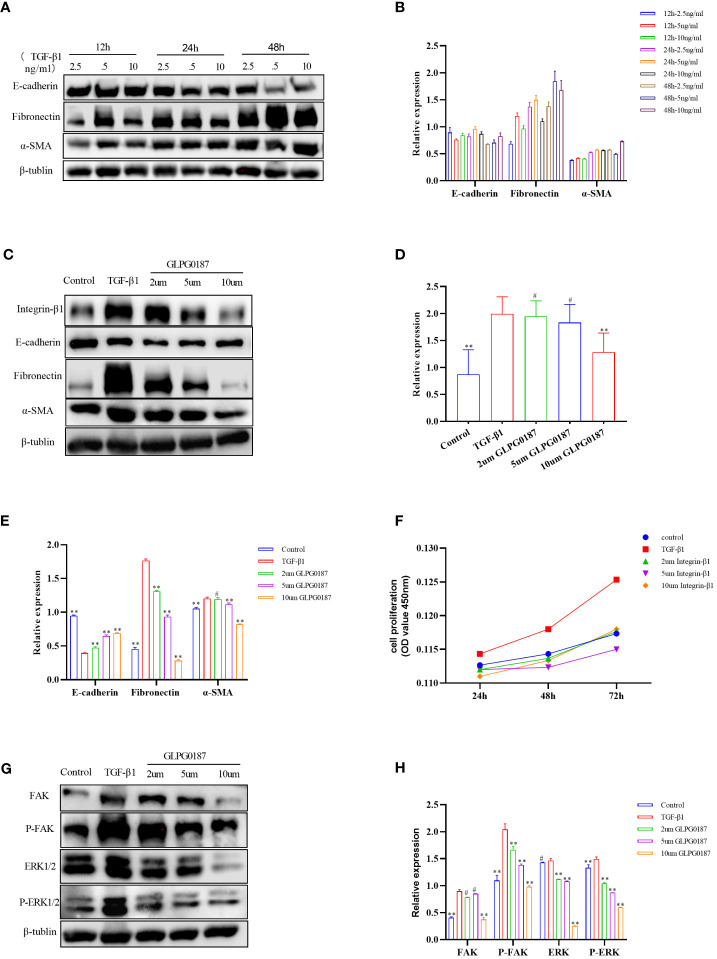
**(A, B)** 5ng/ml of TGF B 1 could induce obvious EMT changes in 16HBE cells. **(C–E)** TGF-β1 upregulated the expression of Integrin-β1, along with the upregulation of a-SMA and fibronectin, but downregulated E-cadherin compared with the control group; GLPG0187 significantly increased the expression of the Integrin-β1, E-cadherin and decreased that of the mesenchymal markers, fibronectin, a-SMA in a concentration-dependent manner. **(F)** GLPG0187 can reduce the 16HBE cells proliferation rate induced by TGF-β1. **(G, H)** TGF-B1 significantly upregulated the expression of the FAK, P-FAK, ERK and P-ERK, while the GLPG0187 can reduce the expression of the above signaling pathway indicators, ^**^P < 0.01, ^#^P>0.05.

The loss of airway epithelial integrity and EMT during AR contributes significantly to asthma pathogenesis (26, 29, 57). To elucidate whether the effects of Sema7a on AR were linked to EMT, 16HBECs were treated with 5 ng/ml TGF-β1, which markedly induced EMT in 16HBECs (**Figures 4A, B**) (58). A previous study reported that Sema7a and its receptor Integrin-β1 contributed to vascular endothelial cell injury and the pathophysiology of atherosclerosis (21, 22, 59). To determine the functional role of Integrin-β1 in the EMT of 16HBECs treated with TGF-β1, the protein expression levels of EMT-related proteins were analyzed using western blotting. As shown in **Figures 4C–E**, compared with the control group, TGF-β1 significantly upregulated the expression of Integrin-β1, α-SMA, and fibronectin but downregulated E-cadherin (*P* < 0.01). Therefore, TGF-β1-induced Sema7a and Integrin-β1 expression may play a critical role in promoting a mesenchymal phenotype.

### 3.5 TGF-β1-induced EMT could be reduced by GLPG0187 in 16HBECs

Integrins are heterodimeric receptors that serve to elicit a series of signal transduction events and sense the extracellular environment that participates in the control of cell cycle progression and apoptosis (α, β) (60). As a member of the integrin subfamily, Integrin-β1 is expressed on T lymphocytes, epithelial cells, and fibroblasts (61–63). The integrin-β1 blocking antibody GLPG0187 was used to detect the interaction between Sema7a and integrin-β1. 16HBECs were treated with different concentrations of GLPG0187 before TGF-β1 treatment to identify the function of Sem7a in EMT in asthma. As shown in **Figures 4C, E**, GLPG0187 significantly increased the expression of E-cadherin and decreased the expression of mesenchymal markers like fibronectin and α-SMA (*P* < 0.01). Additionally, CCK-8 was conducted and showed that proliferation in TGF-β1+ GLPG0187 cells was significantly lower (**Figures 4F**; *P* < 0.01). Taken together, these findings suggest that Sema7a may promote EMT through Integrin-β1 in 16HBECs.

### 3.6 Sema7a may aggravate EMT through the FAK/ERK1/2 signaling pathway in 16HBECs

FAK/ERK1/2 acts as a downstream signal pathway of the Sema7a-integrin-β1 axis, which mediates vascular endothelial dysfunction and tumor metastasis (21, 64). To further characterize the mechanism underlying the promotion of 16HBEC proliferation and migration by Sema7a, the expression levels of FAK, ERK, P-FAK and P-ERK1/2 were assessed using western blotting analysis. As shown in **Figures 4G, H**, GLPG0187 decreased phosphorylated FAK and ERK1/2 levels, suggesting that attenuation of FAK and the ERK1/2 pathway was caused by downregulation of cell surface integrin-β1 (*P* < 0.01). The molecular investigation indicates that Sema7A may promote TGF-β1-mediated EMT, cell migration and proliferation by activating Integrin-β1 and its downstream FAK and ERK1/2 signaling pathways.

## 4 Discussion

This work extends our current understanding of the mechanisms regulating EMT, provides new insights into the role of Sema7a cytokines in bronchial asthma with respect to AR, and strengthens the basis for the Sema7a-integrin-β1 axis in the treatment of human allergic asthmatic EMT and fibrotic diseases. First, we found that the levels of Sema7a in the serum of asthmatic patients were increased (**Figure 1B**). Second, Sema7a was overexpressed in airway and lung tissue in asthmatic mice and promoted airway EMT, which was blocked by an integrin receptor antagonist, GLPG0187. To the best of our knowledge, this is the first study to assess the effect of Sema7a on bronchial epithelial cells in chronic asthma. Finally, GLPG0187 alleviated chronic airway inflammation and AR mainly through the FAK and ERK signaling pathways.

Sema7a is a membrane−associated GPI−linked protein that is mainly expressed on the surface of eosinophils and can also be present in the form of secretion (46, 65, 66). Because eosinophils play an important role in the transmission of airway allergic diseases such as asthma, we hypothesize that Sema7a may be a critical cytokine in the development of allergic airway diseases in asthma (67). First, our study identified, for the first time, the expression of Sema7a in serum and found these to be enhanced in asthma patients (**Figure 1B**; *P* < 0.01). These results are consistent with those of Esnault et al. who found elevated Sema7a levels in asthma patients (45). Previous studies have shown that TGF-β1 is mainly produced by eosinophils accumulated in the peribronchial and perivascular lesions (68). Asthma is characterized by subepithelial fibrosis, and ample evidence exists that eosinophils have a significant pathologic role in promoting airways fibrosis. In a murine fibrosis model, TGF-β1 has been reported to induce the expression of Sema7a in the murine lung (69). One study showed that Sema7a expression is stimulated by TGF-β1 in the murine lung, with Sema7a being a critical regulator of tissue remodeling in TGF-β1-induced pulmonary fibrosis (70). Thus, we speculate that sema7a may promote chronic airway inflammation and AR in asthma. Gan et al. also supported our hypothesis and found that TGF-β1 stimulates fibrocyte accumulation via a Sema7a–dependent, integrin-β1–dependent pathway (65). Our research showed that Sema7a and its receptor integrin-β1 were overexpressed in lung tissue in OVA-treated mice (**Figures 2A–F**; *P* < 0.01); treatment with anti-Sema7a was effective in alleviating AR in the model of chronic asthma. This has been confirmed in the study of Nobuaki Mizutani and Takeshi Nabe (46), in which treatment with anti-sema7a antibody suppressed subepithelial fibrosis in the lungs of Ig-E-sensitized mice. These results suggest that Sema7a is involved in the development of AR by activating its receptor integrin-β1 in asthma.

Epithelium damage and deficiency have been reported to result in EMT, which is considered to be intricately involved in AR and is the main cause of fixed airflow limitations that occur during asthma attacks (26, 71). In contrast, inhibition of the EMT process can alleviate AR in asthma. The increased expression of Sema7a in epithelial cells is closely related to the severity of atherosclerosis (21), and Sema7a has been shown to not only exacerbate inflammation but also promote fibrosis that is associated with EMT (70, 72); in an animal model, the expression of Sema7a in lung tissue was dependent on TGF-β1 (73). The present study supported the findings of the above investigations: integrin-β1 was overexpressed in TGF-β1-stimulated 16HBECs (**Figures 4C, D**, *P* < 0.01); GLPG0187 reduced the expression of integrin-β1 and attenuated the HBEC proliferation and EMT changes induced by TGF-β1 (**Figures 4C–E**, *P* < 0.01). However, the TGF-β1 present in the fetal bovine serum used in the cell culture medium in Stephane Esnault and Elizabeth A. Kelly study does not appear to increase sema7a on eosinophils (74). The inconsistent results of the above studies may be due to the different cell lines. Furthermore, it has been reported that not only integrin-β1 but also Sema7a together with its receptor, plexin C1, regulates cell migration. For instance, the sema7a interaction with plexin C1 expressed on dendritic cells is known to impair their migration (75). However, the interaction between plexin C1 and Sema7a was not further studied in our study; whether Sema7a can inhibit cell migration or EMT by activating plexin C1 remains to be studied. In summary, our results further confirmed the role of the Sema7a-integrin-β1 signaling axis in AR and suggested that it promotes airway EMT and is involved in the pathogenesis of asthmatic AR. Epithelial fibrosis is also an important pathological feature of airway remodeling in asthma. Thus, Sema7a and its integrin-β1 receptor may be potential therapeutic targets for allergic asthma.

The molecular investigation indicated that Sema7a-integrin-β1 and its downstream FAK and ERK1/2 signaling pathways promote nonvascular endothelial growth factor (VEGF) A/vascular endothelial growth factor receptor (VEGFR) 2-mediated cell migration and angiogenesis (23), and FAK/ERK1/2 is involved in T-cell-mediated inflammatory responses (20). Previous studies have shown that Sema7a is not only related to the inflammatory immune response but also regulates axon growth via the mitogen-activated protein kinase (MAPK) signaling pathway (73, 76). ERK constitutes one branch of the MAPK pathway responsible for the invasion of cancer cells by primarily breaking down the extracellular matrix (ECM) (77). A study showed that blockage of integrin-β1 or inhibition of FAK, mitogen-activated protein kinase (MEK) 1/2, or NF-κB significantly reduced the expression of cell adhesion molecules and THP-1 monocyte adhesion in Sema7a-overexpressing human umbilical venous endothelial cells (21). In summary, Sema7a may be involved in asthma airway EMT through the FAK/ERK1/2 signaling pathway. To gain insight into the mechanisms underlying the observed effects of Sema7a, we examined several proteins in the TGF-β1/FAK/ERK/1/2 signaling pathway and identified whether GLPG0187 can affect the kinase activity of FAK and its downstream targets. We analyzed the phosphorylation levels of FAK and ERK1/2 by performing western blotting. Interestingly, the levels of phosphorylated FAK and phosphorylated ERK1/2 were decreased in GLPG0187-pretreated cells (**Figures 4G, H**, *P* < 0.01), suggesting the involvement of FAK/ERK1/2 phosphorylation in the early stage of AR activation.

In conclusion, the present study demonstrated that Sema7a may contribute to asthma AR pathology by affecting HBEC function and airway EMT. GLPG0187 significantly increased the expression of E-cadherin and decreased that of the mesenchymal markers fibronectin and α-SMA in a concentration-dependent manner. These findings may explain the underlying mechanisms of the loss of airway epithelial integrity in OVA-induced asthma by promoting the phosphorylation of FAK and ERK. Collectively, these findings may provide insight into the multifaceted role of Sema7a in chronic asthma. With further studies, Sema7a or integrin-β1 is expected to be used as an immunotherapeutic treatment target for asthma.

Epithelial fibrosis is also an important pathological feature of airway remodeling in asthma. Thus, Sema7a and its integrin-β1 receptor may be potential therapeutic targets for allergic asthma.

## Data availability statement

The original contributions presented in the study are included in the article/**Supplementary Material**. Further inquiries can be directed to the corresponding author.

## Ethics statement

The studies involving human participants were reviewed and approved by Medical Ethics Committee of the First Affiliated Hospital of Shandong First Medical University. The patients/participants provided their written informed consent to participate in this study. The animal study was reviewed and approved by Medical Ethics Committee of the First Affiliated Hospital of Shandong First Medical University. Written informed consent was obtained from the owners for the participation of their animals in this study.

## Author contributions

DL, HP, and FS contributed to the conception and design of the present research. FS and ZG performed the experiments and analyzed the data. HP, YJ, XL, and AZ wrote and revised the manuscript. DL reviewed the article. All authors have read and approved the final version of the manuscript.
