# Supplementary material for: Semaphorin 7a aggravates TGF-β1-induced airway EMT through the FAK/ERK1/2 signaling pathway in asthma
Source: Front Immunol. 2023 Nov 1;14:1167605. doi: 10.3389/fimmu.2023.1167605 (PMC10646317; doi:10.3389/fimmu.2023.1167605)
Supplement: Supplementary file 1 [file Table_1.docx]

<https://www.jianguoyun.com/p/DRPB2e4Q5qe4Cxi1__gEIAA>

<https://www.jianguoyun.com/p/DQSs284Q5qe4Cxi3__gEIAA>

<https://www.jianguoyun.com/p/DX1UxW4Q5qe4Cxi4__gEIAA>

<https://www.jianguoyun.com/p/DWrS9IcQ5qe4Cxi5__gEIAA>

<https://www.jianguoyun.com/p/DVPXUT8Q5qe4Cxi6__gEIAA>

<https://www.jianguoyun.com/p/DdqAVisQ5qe4Cxi8__gEIAA>

<https://www.jianguoyun.com/p/DW7nZ8gQ5qe4Cxi9__gEIAA>

<https://www.jianguoyun.com/p/DUVdNM0Q5qe4Cxi-__gEIAA>

<https://www.jianguoyun.com/p/DajhFL4Q5qe4Cxi___gEIAA>

<https://www.jianguoyun.com/p/DbHQDMAQ5qe4CxjA__gEIAA>

<https://www.jianguoyun.com/p/DTBwycYQ5qe4CxjB__gEIAA>

<https://www.jianguoyun.com/p/DRqHqkgQ5qe4CxjC__gEIAA>

https://www.jianguoyun.com/p/DaU85ygQ5qe4CxjD__gEIAA

<https://www.jianguoyun.com/p/DWp35TQQ5qe4CxjE__gEIAA>
